# Integrating exploration and prediction in computational psychotherapy science: proof of concept

**DOI:** 10.3389/fpsyt.2023.1274764

**Published:** 2024-01-12

**Authors:** Hadar Fisher, Suzannah J. Stone, Sigal Zilcha-Mano, Pavel Goldstein, Timothy Anderson

**Affiliations:** ^1^Department of Psychology, University of Haifa, Haifa, Israel; ^2^Department of Psychology, Ohio University, Athens, OH, United States; ^3^Integrative Pain Laboratory (iPainLab), School of Public Health, University of Haifa, Haifa, Israel

**Keywords:** psychotherapy process and outcome, predictive models, explanatory models, machine learning, alliance, data-driven approach

## Abstract

**Introduction:**

Psychotherapy research has long preferred explanatory over predictive models. As a result, psychotherapy research is currently limited in the variability that can be accounted for in the process and outcome of treatment. The present study is a proof-of-concept approach to psychotherapy science that uses a datadriven approach to achieve robust predictions of the process and outcome of treatment.

**Methods:**

A trial including 65 therapeutic dyads was designed to enable an adequate level of variability in therapist characteristics, overcoming the common problem of restricted range. A mixed-model, data-driven approach with cross-validation machine learning algorithms was used to predict treatment outcome and alliance (within- and between-clients; client- and therapist-rated alliance).

**Results and discussion:**

Based on baseline predictors only, the models explained 52.8% of the variance for out-of-sample prediction in treatment outcome, and 24.1–52.8% in therapeutic alliance. The identified predictors were consistent with previous findings and point to directions for future investigation. Although limited by its sample size, this study serves as proof of the great potential of the presented approach to produce robust predictions regarding the process and outcome of treatment, offering a potential solution to problems such as *p*-hacking and lack of replicability. Findings should be replicated using larger samples and distinct populations and settings.

## 1 Introduction

Traditionally, statistical goals can be divided into two categories: explanation-oriented models, in which researchers are interested in testing an *a priori* hypothesized relationship between two or more variables, and prediction-oriented models, in which researchers are interested in finding an algorithm capable of recognizing which set of variables yields the best predictions about new observations (1, 2). For many years, psychotherapy research focused mainly on models aimed at understanding and explaining associations between variables (3). Many hundreds of studies testing predictors, moderators, and mediators have contributed greatly to our understanding of the process and outcome of psychotherapy (2). For instance, research has shown that the therapeutic alliance is strongly related to improved treatment outcomes, with a more pronounced effect observed in clients dealing with interpersonal problems (4).

Contemporary psychotherapy research is interested not only in driving understanding, but also in making accurate predictions about who may be a good therapist (to direct therapist selection and training efforts), and about treatment prognosis (to select the most efficient treatment for individual clients). Despite the statistical and mathematical similarities, the choice between explanatory models and predictive models can have a significant impact on researchers’ decisions regarding data collection, data preparation, and the statistical models they employ (1). For example, while explanatory models tend to emphasize goodness of fit based on a specific sample and the testing of statistical significance, predictive models assess how accurately the model can apply information about one sample to make correct predictions or decisions when applied to a new dataset (5). Researchers, therefore, must make a deliberate choice to identify the best models for achieving their aims (1, 6).

When following a hypothesis-driven approach, researchers rarely use tools to verify that the models they propose are capable of predicting the outcomes they are modeling (7). Instead, researchers focus largely on the statistical significance of various factors that may be related to the outcome (3). The statistical significance approach does not guarantee predictive accuracy when models are applied to new (i.e., out-of-sample) data. Furthermore, in the past years, there is an increasing concern that many findings in the field cannot be reliably reproduced in subsequent studies, raising questions about the credibility and robustness of psychological research findings (i.e., the replication crisis) (8–11). There is increasing consensus that this widespread replication failure is due largely to “*p*-hacking” and other questionable research practices that have been historically prevalent in the field (12). At the same time, the increased capacity to collect and analyze massive amounts of data, gathered through new technologies or the internet, has contributed to the adoption of a computational approach to analysis (13). While a hypothesis-driven approach is rooted in, and therefore constrained by, substantive theory, a computational approach prioritizes prediction, thus allowing model complexity to increase as long as it continues to enhance predictive performance (7). Inspired by the computational approach, researchers started to use *predictability* models by applying data-driven approaches and principles from the machine learning field to increase the predictive power of the models (7). Machine learning is a subset of artificial intelligence that focuses on developing algorithms and models capable of learning from data and making predictions or decisions without being explicitly programmed. It involves the use of statistical techniques to enable computers to recognize patterns, draw insights, and improve their performance based on experience (5). In this regard, the objectives of machine learning closely parallel those of psychotherapists. Both aim to accumulate knowledge from prior data, such as client histories, and apply that knowledge to new cases, even those that may be unique (14). Machine learning encompasses various approaches, including supervised learning (where models are trained on labeled data), unsupervised learning (for discovering patterns within data), and reinforcement learning (teaching machines to make sequential decisions). These algorithms have applications across a wide range of fields including healthcare, revolutionizing the way we analyze data and solve complex problems (15).

Some promising evidence for the potential utility of implementations of data-driven and machine learning approaches in psychotherapy research has emerged in recent years, answering questions of treatment personalization (16–18) while providing critical solutions to *p*-hacking (19) and replicability issues (10). Moreover, data-driven approaches that use principles from the machine learning field may be better able to capture the richness and complexity of the therapeutic encounter than explanatory models. No single, independent predictor, or even three or four, can be as informative as a set of interrelated variables that jointly increase predictive power to explain variance in the process and outcome of treatment (16). Some of the explanatory approaches, which test each predictor as a separate hypothesis, can lead to erroneous conclusions because of multiple comparisons (inflated type I errors), model misspecification, and multicollinearity (20). Findings may also be affected by publication bias, as statistically significant predictors have a better chance of being reported in the literature (21). Machine learning approaches provide a robust solution to these problems by looking at data insights, developing a predictive model, and finally, applying self-validation procedures (22, 23). However, a significant drawback of some (but not all) machine learning approaches is their tendency to treat models mostly as a “black box,” resulting in a lack of interpretability of the findings (3, 7).

We sought to integrate the benefits of the interpretability and predictability of statistical models. As such, the present study was designed as a proof of concept of the ability of models to predict variability in treatment process and outcome, based on pre-treatment client and therapist characteristics. Our study focused on changes in psychological dysfunction from pre- to post-treatment as the treatment outcome. We chose the therapeutic alliance as an example of a process variable because it is one of the most researched constructs in psychotherapy research, and one of the most consistent predictors of treatment outcome (24). Two important aspects of the alliance are of interest when predicting clients’ and therapists’ reported alliance: (a) the trait-like tendency of the client and therapist to form a strong alliance across various phases of treatment (also known as the between-clients alliance), and (b) the state-like changes occurring during treatment in client and therapist reports of the alliance (the within-client alliance). The sample was chosen to avoid restriction of range, a substantial risk when testing therapist characteristics because in many trials only a narrow range of therapist characteristics is allowed. As the therapist effect may have been skewed in previous studies owing to restriction of range, to increase variance, we selected therapists prospectively, based on their level of social skills and performance on a simulated task. For the same reason, we also selected both trained and untrained therapists. Using prediction models in a sample that is less restricted in range, we expect the baseline characteristics of clients and therapists to predict a large portion of treatment outcome and within- and between-clients alliance.

For client characteristics that may affect the alliance and the outcome of treatment, we focused on those that have received extensive attention in the literature. Specifically, we focused on clients’ demographics (gender, age, and income), symptom severity, and functioning. We also focused on clients’ interpersonal skills and problems [for a review, see (25, 26)]. Whereas previous studies showed little evidence that demographic variables significantly predict psychotherapy outcome or alliance, more complex results were found concerning symptom and interpersonal problem severity (25). Severity of symptoms and functional impairment predicted worse outcome and lower trait-like alliance [e.g., (27–30)]. At the same time, severity of symptoms also predicted greater improvement of both symptoms and state-like alliance [e.g., (31–33)]. The same pattern of results was found for interpersonal skills and problems [e.g., (34–36)].

With regard to therapists, in addition to including therapist training and clinical orientation as potential predictors of treatment process and outcome, we focused on therapist characteristics that are typically easily available in clinical practice, including demographics (age, gender, and income), and on those receiving theoretical and empirical support, such as previously being in treatment themselves (37), and Facilitative Interpersonal Skills [FIS; (38)]. Previous studies examining the association between such therapists’ characteristics and alliance or outcome have yielded mixed results. Specifically, whereas previous studies mostly found that training and experience were not related to outcome and alliance [e.g., (39–41)], other studies found them to be related to poorer outcome and lower alliance (42–44). Manne et al. (45) reported that therapists’ experience negatively predicted mean alliance when alliance was rated by the client, but positively predicted alliance when alliance was rated by the therapist. These findings may suggest that a complex predictive model is needed to better explain the mixed results.

In the same vein, most studies examining the relationship between the therapist’s demographic variables and alliance or treatment outcome have found that these variables are not significantly related to therapy outcome or alliance [Outcome: (37, 46, 47); Alliance: (42, 45)]. At the same time, other studies suggested that therapists being older or a woman may be related to outcome and alliance, presumably because of their relation to superior interpersonal and social skills (48). For instance, results of a large naturalistic study suggested that gender may have an indirect role on alliance and outcome such that female therapists intervene more empathically, whereas male therapists tend to use more confrontational techniques (49). Similarly, Anderson et al. (38) found that older therapists produced superior outcomes. However, when therapists’ social skills and FIS were examined, age no longer predicted outcome. These results may point to the importance of including both demographic characteristics and interpersonal and social skills in the predictive model for better prediction of alliance and outcome. One way to measure therapists’ interpersonal and social skills is using the FIS. FIS includes common relational skills, such as the ability to respond empathetically to clients, express the appropriate amount and type of emotion when responding to clients, and efficiently respond to ruptures that may arise in the alliance with clients. FIS is measured using a performance task in which therapists record audio responses to a set of stimulus clips simulating various difficult interpersonal situations derived from real therapy sessions. Each therapist’s responses are then rated by trained observers for their coverage of the eight FIS items: verbal fluency, emotional expression, persuasiveness, warmth/acceptance/understanding, hope/positive expectations, empathy, alliance bond capacity, and alliance-rupture-repair responsiveness (38, 50). Therapist FIS has been found to predict treatment outcome across settings (38, 51, 52). Anderson et al. (28) found that therapist FIS predicted outcomes at a university counseling center. In a randomized clinical trial, Anderson et al. (51) found that helpers with significantly higher FIS showed better outcomes and developed stronger client-related alliances when counseling undergraduate students with heightened levels of clinical distress. In a naturalistic study, Anderson et al. (42) found that therapist FIS predicted the outcomes of clients treated by graduate students in clinical psychology over 1 year later.

In addition to FIS’s prediction of outcome in several studies, multiple metanalyses have revealed several other predictors of outcome [e.g., (2)]. For example, a previous review by Beutler (53) identified multiple therapist characteristics, such as their training and experience, which give promise to predicting outcome. Nevertheless, at that point, these characteristics were impossible to consider jointly. It is reasonable to include these client and therapist characteristics in a model which allows them to combine with FIS. Furthermore, the notion of therapist responsiveness (54) recognizes that there are a very large number of possible variables within the “emergent context” (55) that might be accounted for to explain what works in any particular therapeutic situation.

Taken together, in the present study, we aim to benefit from the advantages of the two potentially complementary approaches, the interpretability and predictability approaches. We will do so by applying a data-driven approach and principles from the multilevel machine learning framework. Our approach allows: (a) identifying the models with the best fit to the data (given a set of potential predictors), considering the linear and quadratic shapes of the relationships as well as pairwise interactions, and taking into account their nested data structure; (b) providing an exact interpretation of all model effects; (c) cross-validating the proposed model; and (d) estimating the contribution of each model effect, as defined below.

## 2 Methods

### 2.1 Participants

The clients were 65 individuals who showed significant distress and met criteria for a DSM-IV psychological disorder, selected to participate in the study from 2,713 undergraduates. The participants were selected on the basis of significantly high scores (i.e., at least two standard deviations above the mean) across two administrations of a general symptom measure as well as a brief assessment interview. For more information about client selection procedure, see Anderson et al. (51). For ethical considerations, given the diversity of training and experience of the individuals playing the role of therapists in this trial, none of the participants who were recruited for the study were actively seeking clinical services at the time. Of the 65 individuals who completed the study as clients, 64 provided demographic data. Thirty-five clients (54.7%) identified as female, while 29 (45.3%) identified as male. Most of the sample was White (*n* = 60, 93.8%). Other clients self-identified their race/ethnicity as Asian/Pacific Islander (*n* = 1, 1.6%), Black (*n* = 1, 1.6%), and Hispanic (*n* = 2, 3.1%). The average client age was 19.2 (*SD* = 1.1). Diagnostic groupings were as follows: adjustment disorder (*n* = 12, 18.8%), dysthymia (*n* = 15, 23.4%), generalized anxiety (*n* = 14, 21.9%), major depression (*n* = 10, 15.6%), personality (*n* = 5, 7.8%), and miscellaneous (e.g., phobia, panic, eating; *n* = 8, 12.5%).

The therapists were 23 (eight male, 15 female) doctoral students, selected from a larger group of 56 applicants. Therapists were selected with the intention of creating independent groups on the basis of their interpersonal skills (i.e., low vs. high) and training status (i.e., trained vs. untrained). Low or high interpersonal skills was defined by scores on a self-report measure of social skills. The individuals that were selected as therapists for this study were taken from the highest and lowest quarter of performances on a social skills measure. In other words, those who deviated most from the (gender-specific) mean. For more information about skill selection procedure, see Anderson et al. (51). For training status, 11 therapists were in a clinical psychology doctoral program and had completed at least 2 years of training; these were considered the “trained” group. The other 12 therapists, constituting the “untrained” group, had no clinical or psychotherapeutic training but had completed at least 2 years in doctoral programs in various other disciplines. Therapists’ ages ranged from 23 to 53 years (mean = 30.61 years; *SD* = 9.32). Therapists self-identified as 83% Caucasian, 13% Asian, and 4% Hispanic. Each therapist worked with two clients.

### 2.2 Measures

#### 2.2.1 Outcome questionnaire-45

The Outcome Questionnaire-45 (OQ-45) is a 45-item general symptom measure that was completed by clients (56). The items assess the following three primary dimensions: (a) subjective discomfort (e.g., anxiety and depression), (b) interpersonal relationships, and (c) social role performance. Each item is rated on a five-point Likert scale, ranging from 0 (never) to 4 (almost always). The sum of the items (after reverse-coding selected items) forms the total OQ-45 score, which was used in the current study. The measure has demonstrated good internal consistency in prior studies [*α*_s_ ranging from 0.70 to 0.93; (57)], as well as in the current study (α = 0.96). In case of missing data, we followed the standard scoring rules, according to which only forms with fewer than four missing items were included in the following analysis.

#### 2.2.2 Inventory of interpersonal problems

The Inventory of interpersonal problems (IIP-64) is a measure of interpersonal distress commonly used for measuring treatment changes in the interpersonal domain (58). The degree of distress associated with each item is rated on a five-point scale, ranging from 1 (not at all) to 5 (extremely). Test–retest reliability for the IIP for a 10-week period has been reported at 0.98 for the overall inventory, with internal consistency ranging from 0.82 to 0.93 (59). The present sample had good internal consistency as well (*α* = 0.93 at pretreatment and termination and *α* = 0.95 at 3-month follow-up).

#### 2.2.3 Social skills inventory

The social skills inventory (SSI) is a 90-item self-report questionnaire that assessed self-reported social skills (60). Items were scored using five-point Likert scaling, from 1 = “not at all like me” to 5 = “exactly like me.” The SSI measures skills in expressivity, sensitivity, and control in verbal (social) and non-verbal (emotional) domains. The total of the items provided an overall indicator of social skills, which was used in this study. The scale has high internal consistency, and factor analytic studies have supported the multidimensional structure of the scale. Coefficient alphas range from 0.75 to 0.88. Test–retest correlations range from 0.81 to 0.96 for a 2-week interval, and alpha coefficients range from 0.62 to 0.87 (60). Convergent and discriminant validity for the SSI were supported in a series of studies conducted by Riggio (60). In the present study, the SSI was completed by both therapists (as a selection variable) and clients in the study (at pre-treatment). The SSI had good internal consistency in the present sample (*α* = 0.88).

#### 2.2.4 Facilitative interpersonal skills

Facilitative interpersonal skills (FIS) is an observational rating of audio responses provided by therapists to difficult simulated clients (38, 51). There are eight items on the rating scale, all of which pertain to the therapist’s skill in fostering facilitative conditions. These eight skill domains are verbal fluency, emotional expression, persuasiveness, warmth/positive regard, hopefulness, empathy, alliance bond capacity, and alliance-rupture-repair responsiveness. Each of these domains was rated on a five-point Likert scale, ranging from 1 (skill deficit) to 5 (optimal presence of the skill). All ratings were initially anchored at 3 and were moved up or down the scale based on evidence of skills found in the audio responses. To increase reliability of these ratings, a coding manual was used that provided descriptions for each of the skills. The FIS ratings were made by four coders, which included one doctoral-level researcher (Caucasian male), two graduate students (Chinese female and Caucasian male), and one undergraduate student (Caucasian female). Instruction in the FIS rating method occurred weekly over a 2-month period. Once there appeared to be sufficient agreement, ratings for the study commenced. The prospective therapist responses were rated in random order and in sets of 10. Each coder made their ratings separately and independently. Calibration meetings were held after each set of ratings, where discussion focused on those ratings that were most discrepant (i.e., typically greater than 1 point discrepancy). Final ratings for analysis in the study were a mean of all eight items, which were averaged across the four coders. Interrater reliability was acceptable for total FIS (intraclass correlation coefficient = 0.86), and the internal consistency of the eight FIS items was high (α = 0.96).

#### 2.2.5 Working alliance inventory (WAI-C and WAI-T)

The WAI is a 36-item measure of the quality of the therapeutic alliance (61). It contains subscales for measuring agreement on tasks, goals, and the existence of a therapeutic bond. Each subscale contains 12 items, which the participant rated on a seven-point Likert type scale, ranging from 1 (never) to 7 (always). Across all sessions, the WAI-C, administered to clients, had good internal consistency with alpha ranging from a low of 0.79 to a high of 0.90, and the WAI-T, administered to therapists, ranging from α = 0.80 to 0.81.

### 2.3 Procedure

Study procedures are described in a previous report (51). Selected therapists were randomly assigned to two clients each. After describing the study to the clients, written informed consent was obtained. Clients were given the opportunity to receive a referral for treatment elsewhere and were also notified that they may discontinue their involvement in the study at any time. Clients were told that they would meet with a “helper” for sessions, who would try to aid them with their problems. At the first session, clients received brief instructions to discuss their problems with the “helper.” Treatments lasted 7 weeks, one session per week. No clients chose to discontinue their involvement in the study at any time.

Treatment outcome was measured pre-treatment, at sessions 1, 3, 5, and 7, and post-termination, using the OQ-45 (56). Alliance was measured at sessions 1, 3, 5, and 7, using the WAI (61). Baseline predictors included: clients’ and therapists’ demographics, clients’ IIP-64 (58), clients’ SSI (60), therapists’ FIS (51, 52), and the OQ-45 total score and subscales. All methods were carried out in accordance with relevant guidelines and regulations. The study was approved by the Institutional Review Board at Ohio University and all ethical standards were followed.

### 2.4 Statistical analyses

#### 2.4.1 Identifying the models with the best fit to the data

Model selection is a crucial step in data-driven modeling. In both linear and nonlinear modeling, selection criteria are generally used to identify a model that (a) fits the data well, (b) consists of model variables that can be easily interpreted, (c) involves a parsimonious representation, and (d) can be used for inference and model prediction. In the present study, we applied a search for the best subset of input parameters, considering all possible models that consist of up to nine terms: linear and quadratic variable effects, and pairwise interactions. To account for the nested structure of the data (therapist and client levels), we used multilevel models with the lmer function of the R package lme4 (62). This package is commonly employed by researchers for testing hypotheses in multilevel treatment data. However, by utilizing a data-driven search for the best model—a principle from the machine learning framework—we not only enhance the validity of model inferences but also bolster predictive power. Simultaneously, this process enhances the interpretability of the findings.

#### 2.4.2 Training and cross-validation

We systematically searched for the best subset of input parameters, considering all possible models that consist of up to nine terms, due to the limited sample size: linear, quadratic effects, and pairwise interactions. To take into account the nested data structure, we applied multilevel models using the lmer function of the R package lme4 (62) inside the machine learning framework. The best model was selected based on the Akaike Information Criterion (AIC) (63) and cross-validation. Specifically, we employed leave-one-out cross-validation (LOOCV) to identify a model with the lowest AIC in the training phase while ensuring that the explained variance (*R*^2^) for the LOOCV did not decrease by more than 10%, which would indicate potential overfitting. Using LOOCV in model selection provides advantages such as robust evaluation by testing on multiple validation sets. Minimizing the AIC helps find a balanced model fit while considering complexity. LOOCV builds a model for each data point, creating as many models as data points. It leaves one data point out for validation in each iteration, training the model on the rest. The term “training” refers to the process of teaching the model to make predictions based on the dataset. During training, the model adjusts its parameters and internal settings using the training data to understand patterns and relationships. After training, the model can make accurate predictions or classifications when given new, unseen data. In LOOCV, model results are combined, typically by averaging or summing, to assess overall performance and generalization. It is recommended for smaller datasets but can be computationally expensive for large ones. LOOCV does not involve splitting data into traditional training and test sets; each model uses one data point as a test while others are for training, repeating for all data points (64). We opted LOOCV over the more conventional 10-fold cross-validation for several reasons. First, our dataset is relatively small, and with this limited data, LOOCV is less prone to overfitting as it employs nearly all available data for training in each iteration. Secondly, LOOCV typically yields less biased estimates of model performance, particularly in cases of limited data, which is essential for obtaining a precise evaluation of our model’s capabilities. Lastly, LOOCV maximizes data utilization by ensuring that every data point is used for both training and testing, which is particularly advantageous when working with a small dataset, allowing for a comprehensive understanding of the model’s behavior (65, 66). However, it should be noted that LOOVC runs the risk of overestimating prediction accuracy, or conversely, underestimating prediction error. We report model effect size based on the quasi-*R*^2^, as proposed by Nakagawa and Schielzeth (67).

#### 2.4.3 Providing exact interpretation of all model effects

**Figure 1:**
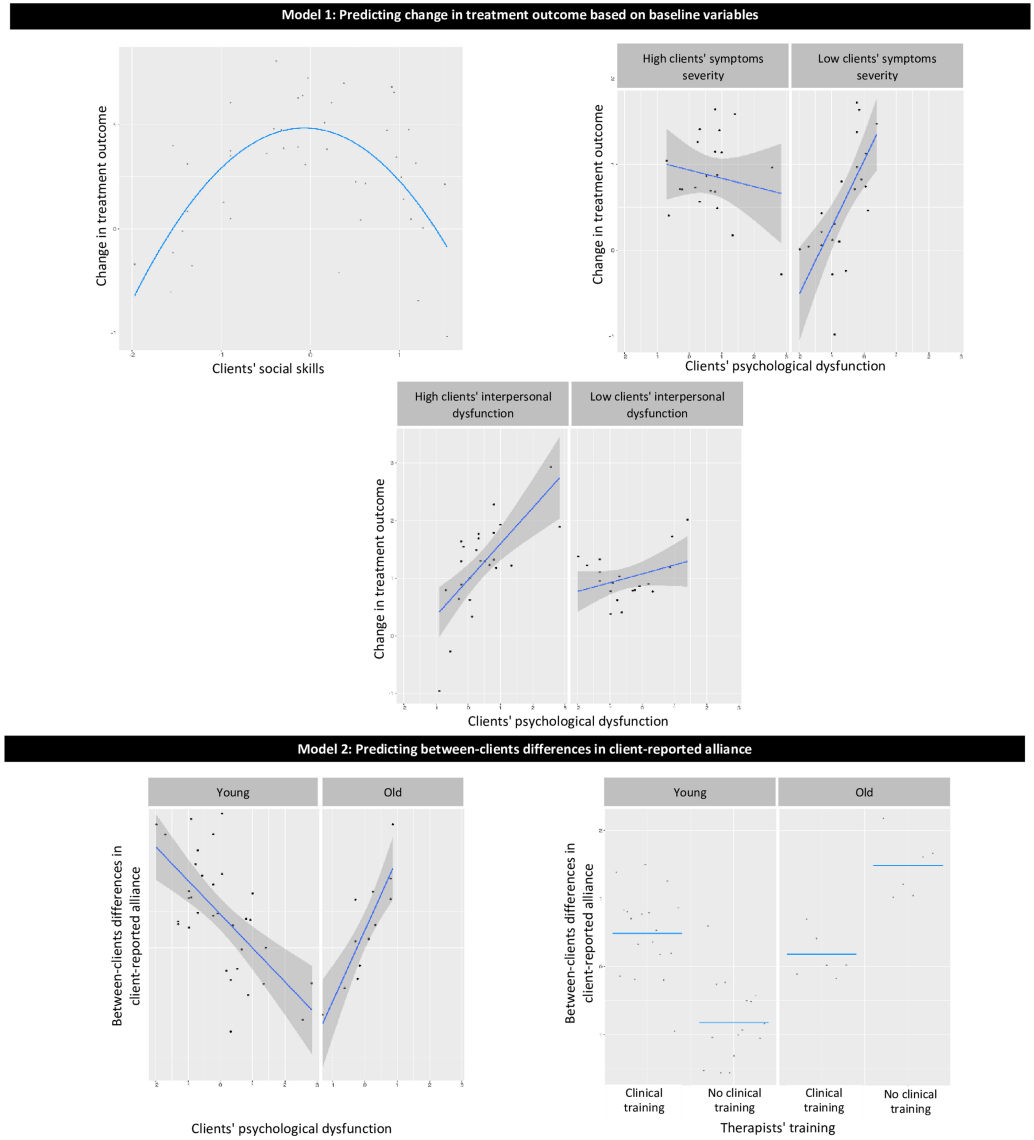
Graphical presentation of the effects in models 1–2. Model 1: predicting change in treatment outcome based on baseline variables; Model 2: predicting between-client differences in client-reported alliance.

**Figure 2:**
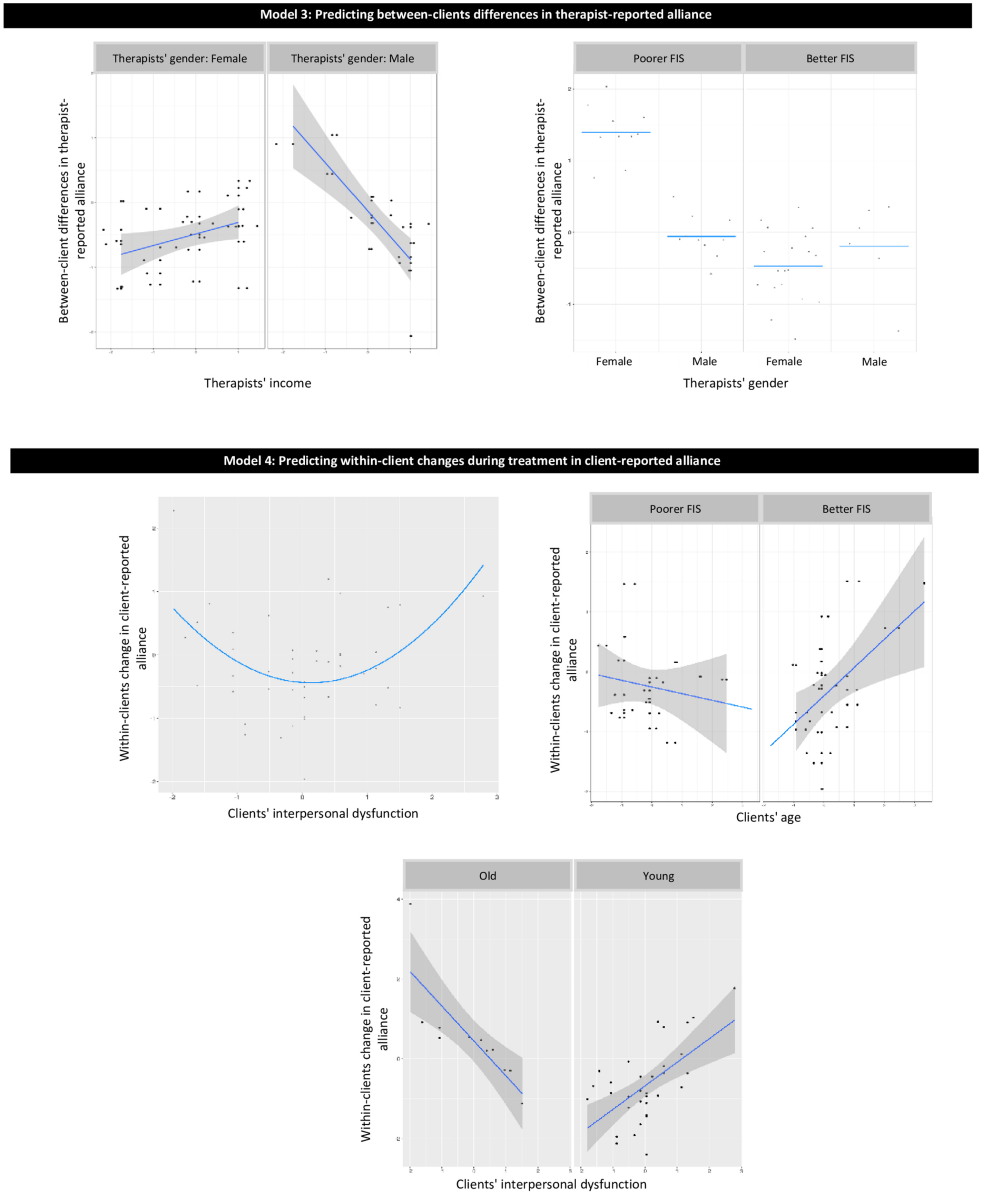
Graphical presentation of the effects in models 3–4. Model 3: predicting between-client differences in therapist-reported alliance; Model 4: predicting within-client changes during treatment in client-reported alliance.

**Figure 3:**
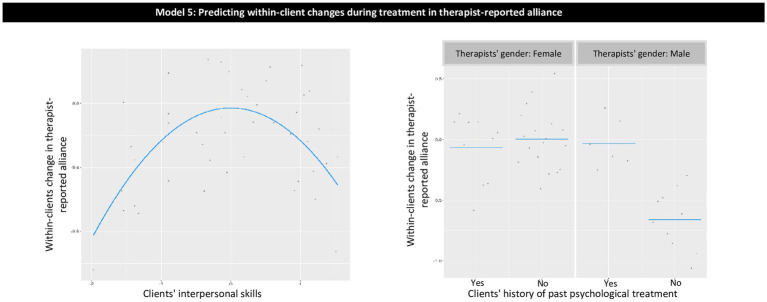
Model 5: predicting within-client changes during treatment in therapist-reported alliance.

The proposed restriction for the potential model effects (described above) also helps to filter out complicated effects with a low level of interpretability. Linear effects are easy to interpret merely based on model coefficients. For quadratic effects and pairwise interactions, we provided plots of the effects to increase their interpretability (Figures 1–3).

We used this approach to predict five client-level psychotherapy process and outcome variables. We used Model 1 to identify the best model for predicting change in treatment outcome. We operationalized change in treatment outcome as the client-specific slope of psychological dysfunction from pre- to post-treatment. Model 2 served to predict between-clients differences in the client-reported alliance, and Model 3 in the therapist-reported alliance. In Models 2 and 3, the aggregated level of alliance across time served as an estimation of between-clients alliance. Model 4 served to predict within-client changes in the client-reported alliance of the course of treatment. Model 5 served to predict within-client changes in the therapist-reported alliance. In Models 4 and 5, the client-level session-related random slopes (time effect), calculated based on the mixed models, served as an estimation of the within-client alliance.

In all models, pre-treatment client and therapist characteristics were used as predictors. We used the following predictors: client and therapist demographics (gender, age, and income), therapist characteristics (FIS, clinical orientation, and whether they had clinical training), client characteristics (Inventory of Interpersonal Problems, Social Skills Inventory, Outcome Questionnaire at baseline, and its three subscales, and whether they had treatment before). All continuous predictors were preprocessed by *Z*-score transformation, and all binary predictors were scored as 0.5 vs. −0.5. Four missing values in income were imputed using the k-nearest neighbor imputation approach (68). We tested normality assumption and heteroscedacity of the residuals in the final models. The assumptions were met. K-nearest neighbor imputation is performed by finding the k closest samples (Euclidean distance) in the training set. Imputation by bagging fits a bagged tree model for each predictor, as a function of all the others (69). KNN-based imputation demonstrated an adequate performance compared to other imputation approaches though using only a single imputed dataset may underestimates the standard error estimations. (70).

A data-driven approach was used to produce predictions in a rigorous test to increase the predictive power of the models, enhance the replicability of the findings, and enable the interpretation of each effect. We focused only on models that can be replicated outside the sub-sample used to build the models. Unlike many machine learning approaches, this one accounted for the nested structure of the data (clients nested within therapists) and provided interpretable results, rather than findings in a “black box” format.

## 3 Results

### 3.1 Model 1: predicting change in treatment outcome based on baseline variables

The model explained 52.8% of the variance in treatment outcome for out-of-sample prediction. Variables contributing to the explained variance were as follows (Table 1; Figure 1, Model 1): (a) therapists’ cognitive, dynamic, and humanistic orientations were related to less reduction in symptoms than were eclectic or undetected orientation; (b) intermediate level of client social skills predicted less symptom reduction than did low or high client social skills; (c) for clients with lower levels of baseline symptom severity, more severe psychological dysfunction was associated with less symptom reduction, whereas for clients with higher levels of baseline symptom severity, no significant association was found between psychological dysfunction and symptom reduction; and (d) for clients with more interpersonal dysfunction, more psychological dysfunction was associated with less symptom reduction, whereas for clients with lower levels of interpersonal dysfunction, no significant association was found between psychological dysfunction and symptom reduction.

**Table 1:** A predictive model of treatment outcome.

Predictors	Estimates	CI	df	Statistic	*p*
(Intercept)	0.22	−0.02 to 0.46	31	1.76	0.094
Therapeutic orientation	−0.42	−0.65 to −0.19	31	−3.60	**0.002**
Interpersonal problems	0.20	−0.02 to 0.42	31	1.78	0.084
Client’s social skills	0.24	−1.12 to 1.59	31	0.34	0.735
Client’s social skills^2^	−2.64	−4.06 to −1.22	31	−3.64	**0.001**
Client’s symptom severity	−0.08	−0.78 to 0.63	31	−0.21	0.833
Client’s psychological dysfunction	0.35	−0.56 – 1.26	31	0.75	0.459
Client’s interpersonal dysfunction	−0.05	−0.49 to 0.39	31	−0.21	0.831
Client’s symptom severity × Client’s psychological dysfunction	−0.45	−0.65 to −0.26	31	−4.56	**<0.001**
Client’s interpersonal dysfunction × Client’s psychological dysfunction	0.27	0.07–0.48	31	2.61	**0.013**
Random effects					
σ^2^	0.35				
τ_00 tid_	0.07				
ICC	0.17				
Marginal training/CV *R*^2^	0.613/0.528				

CV, Cross validation; Interpersonal dysfunction, Interpersonal relationship subscale of the Outcome Questionnaire; Interpersonal problems, Total score of the IIP scale; psychological dysfunction, Outcome questionnaire total score; Symptom severity, Symptoms (e.g., anxiety and depression) subscale of the outcome questionnaire. ^2^refer to quadratic effects. Significant *p*-values are in bold.

### 3.2 Model 2: predicting between-clients differences in client-reported alliance

The model explained 32.2% of the variance for out-of-sample prediction of between-clients differences in client-reported alliance. Variables contributing to the explained variance were as follows (Table 2; Figure 1, Model 2): (a) interaction between age and psychological dysfunction such that for younger therapists, more psychological dysfunction was associated with a weaker alliance, whereas for older therapists, more psychological dysfunction was associated with a stronger alliance; (b) interaction between age and clinical training such that for younger therapists, those with clinical training had a stronger alliance than those without clinical training, whereas, for older therapists, those with clinical training had a weaker alliance than those without clinical training.

**Table 2:** Model 2: predicting between-clients differences in client-reported alliance.

*Predictors*	*Estimates*	*CI*	*df*	*Statistic*	*p*
(Intercept)	0.03	−0.28 to 0.34	32	0.18	0.856
Therapist age	0.27	−0.17 to 0.72	32	1.21	0.243
Psychological dysfunction	0.29	0.02–0.55	32	2.14	0.041
Training (yes)	−0.00	−0.32 to 0.32	32	−0.01	0.989
Therapist gender (male)	0.15	−0.16 to 0.46	32	0.95	0.359
Client gender (male)	0.27	0.01–0.52	32	2.03	0.052
Therapist age × Psychological dysfunction	0.76	0.33–1.19	32	3.45	**0.002**
Therapist age × Training (yes)	0.66	0.20–1.12	32	2.79	**0.013**
Therapist age × Therapist gender (male)	0.34	−0.03 to 0.70	32	1.81	0.088
Random effects					
σ^2^	0.48				
τ_00 tid_	0.28				
ICC	0.36				
Marginal training/CV *R*^2^	0.374/0.322				

Significant *p*-values are in bold.

### 3.3 Model 3: predicting between-clients differences in therapist-reported alliance

The model explained 52.8% of the variance for out-of-sample prediction of between-clients differences in therapist-reported alliance. Variables contributing to the explained variance were as follows (Table 3; Figure 2, Model 3): (a) for poorer FIS, female therapists showed stronger alliance than did male therapists, whereas for better FIS, no significant differences were found between males and females; and (b) for males, higher therapist income was associated with weaker alliance, whereas for females, there was no significant association between income and alliance.

**Table 3:** Model 3: predicting between-clients differences in therapist-reported alliance.

Predictors	Estimates	CI	df	Statistic	*p*
(Intercept)	1.38	0.87–1.89	35	5.28	<0.001
Therapist gender (male)	−1.37	−2.08 to −0.66	35	−3.78	0.001
FIS (low)	−1.86	−2.49 to −1.23	35	−5.81	<0.001
Therapist income	0.18	−0.04 to 0.39	35	1.64	0.114
Therapist gender (male) × FIS	1.72	0.66–2.79	35	3.18	**0.005**
Therapist gender (male) × Therapist income	−0.92	−1.39 to −0.46	35	−3.89	**<0.001**
Random effects					
σ^2^	0.29				
τ_00 tid_	0.17				
ICC	0.37				
Marginal training/CV *R*^2^	0.582/0.528				

Significant *p*-values are in bold.

### 3.4 Model 4: predicting within-client changes during treatment in client-reported alliance

The model explained 43.8% of the variance for out-of-sample prediction of within-client changes in client-reported alliance. Variables contributing to the explained variance were as follows (Table 4; Figure 2, Model 4): (a) intermediate levels of interpersonal dysfunction were associated with less strengthening of the alliance throughout the course of treatment; (b) for better FIS, older clients showed more within-client strengthening of the alliance than did younger ones, whereas for poorer FIS, there was no association between client age and alliance; and (c) for younger clients, clients’ interpersonal dysfunction did not predict alliance, whereas, for older clients, higher interpersonal dysfunction was associated with less strengthening of within-client alliance.

**Table 4:** Model 4: predicting within-client changes during treatment in client-reported alliance.

Predictors	Estimates	CI	df	Statistic	*p*
(Intercept)	−0.08	−0.29 to 0.14	34	−0.68	0.501
Interpersonal dysfunction	−0.40	−1.89 to 1.09	34	−0.53	0.600
Interpersonal dysfunction^2^	2.48	0.71–4.25	34	2.75	**0.009**
FIS (low)	−0.07	−0.30 to 0.16	34	−0.63	0.534
Client age	0.23	−0.02 to 0.48	34	1.81	0.079
FIS × Client age	0.29	0.06–0.52	34	2.50	**0.017**
Client age × Interpersonal dysfunction	−0.50	−0.88 to −0.12	34	−2.60	**0.013**
Random effects					
σ^2^	0.53				
τ_00 tid_	0.00				
Marginal training/CV *R*^2^	0.506/0.438				

^2^refer to quadratic effects. Significant *p*-values are in bold.

### 3.5 Model 5: predicting within-client changes during treatment In therapist-reported alliance

The model explained 24.1% of the variance for out-of-sample prediction of within-client changes in therapist-reported alliance. Variables contributing to the explained variance were as follows (Table 5; Figure 3): (a) intermediate levels of clients’ interpersonal skills predicted more strengthening in within-client alliance than did low or high levels of clients’ interpersonal skills; and (b) for male therapists, a history of previous psychological treatment predicted more strengthening in within-client alliance than no history of previous treatment, whereas, for females therapists, there was no significant association between history of previous psychological treatment and alliance.

**Table 5:** Model 5: predicting within-client changes during treatment in therapist-reported alliance.

Predictors	Estimates	CI	df	Statistic	*p*
(Intercept)	0.72	−0.00 to 1.44	32	1.96	0.062
Interpersonal dysfunction	0.24	0.09–0.40	32	3.06	0.006
FIS (low)	−0.83	−1.59 to −0.07	32	−2.15	0.044
Client interpersonal skills^2^	−0.20	−0.39 to −0.01	32	−2.11	**0.046**
Therapist gender (male)	0.05	−0.83 to 0.94	32	0.12	0.909
Client history of past psychological treatment	0.06	−0.33 to 0.46	32	0.32	0.755
Therapist gender × Client history of past psychological treatment	−0.71	−1.37 to −0.05	32	−2.11	**0.047**
Random effects					
σ^2^	0.14				
τ_00 tid_	0.69				
ICC	0.83				
Marginal training/CV *R*^2^	0.301/0.241				

^2^refer to quadratic effects. Significant *p*-values are in bold.

The R code and further information regarding the models employed in this study can be accessed at https://osf.io/uzjhy/. Additionally, model evaluation metrics and correlation metrics between the study variables are provided in the supplement.

## 4 Discussion

Results of the current study showed that a large portion of variance, both in alliance and outcome, can be explained based only on the pre-treatment characteristics of clients and therapists. Baseline characteristics (clients’ psychological dysfunction, interpersonal dysfunction, and social skills, and therapists’ general treatment orientation) were able to predict 52.8% of the variance in outcome and 24.1–52.8% of the variance in alliance for clients whose data were not used for building the models on which the predictions were based, to avoid overfitting. Thus, the present study demonstrates the potential utility of integrating explanatory and predictive models for psychotherapy science. Such integration may allow for deriving theoretical insights through selecting the variables that have the most relevance in terms of explanation (based on the theoretical framework) and including them in a model of prediction of treatment processes and outcomes. Whereas the most prominent explanatory studies in psychotherapy today can explain about 5–7% variance (71), the present study increased this range several fold.

Results of the current study showed that changes in symptoms from pre- to post-treatment were predicted mostly by clients’ baseline symptoms as well as psychological and interpersonal functioning. Previous studies using machine learning to predict treatment response found mixed results. For example, whereas Yin et al. (72) reported that greater baseline symptoms severity were among the most important predictors of treatment response, Ziobrowski et al. (73) did not find baseline symptom severity to be among the important predictors. Future studies should address this discrepancy in the literature. If replicated, the finding that baseline symptoms predict treatment response may suggest that in tailoring treatment to the individual client, considerable attention should be given to the clients’ symptomatic complaints (25, 26). The process of tailoring treatment to the individual client can be further enhanced using network analysis to recognize the key symptoms and their dynamics when identifying treatment targets (74, 75).

Concerning alliance prediction models, using both within- and between-clients prediction enabled us to recognize predictors of both state-like and trait-like components of the alliance. At the trait-like component, we found that therapist characteristics were better predictors of trait-like alliance than were client characteristics. Specifically, we found that therapist age moderated the effect of training on alliance, with training being associated with better alliance only for young therapists. In addition, we found that therapists’ gender moderated the association between therapists’ income and alliance, with higher income being associated with poorer alliance only in male therapists. Gender also moderated the association between FIS and alliance, with poorer FIS being associated with poorer alliance only in male therapists. Considering this proof-of-concept study, we will not individually elaborate on each outcome. However, in a broader sense, the observed interaction effects potentially indicate that cultural factors might influence how therapists’ characteristics impact the therapeutic alliance. For instance, income could affect males differently, implying that financial status might correlate with the therapeutic relationship in gender-specific ways. Moreover, interpersonal skills, often more culturally encouraged in women who tend to exhibit greater supportive communication, potentially leading to a reduction in the variance explained by FIS among women (76). In our sample, 66% of the females had high FIS scores, whereas only 37.5% of the men had high FIS scores. This is in accordance with a recent study indicating that female students demonstrate superior interpersonal skills compared to males in the context of medical consultations (77). Importantly, the centrality of therapists’ characteristics in predicting alliance is consistent with a recent meta-analysis by Del Re et al. (78), who found support for therapist effects on alliance, indicating that some therapists are better at forming strong alliances with their clients than others. Still, previous studies focused mainly on a given therapist’s characteristics at a certain point in time. For example, one study found that therapist age was positively associated with trait-like alliance but found no effect of gender or therapist years of experience on alliance (42). Using the proposed framework, we were able to identify a more nuanced understanding that has the potential to elucidate previously inconsistent findings in psychotherapy research.

The current findings, together with those reported in the literature (79–82), highlight the importance of therapist characteristics in determining alliance and treatment outcome. Such characteristics, when supported by theory and clinical observations, can be used to identify candidates for clinical training who are expected to form strong alliances with their clients. Furthermore, characteristics that are amenable to change and will be found to be causally related to outcome should be the focus of empirically guided training programs for clinicians (83).

The findings suggest that whereas trait-like alliance was predicted mostly by therapist characteristics, state-like changes in alliance over the course of treatment were predicted by both client and therapist characteristics, and by the interaction between them. Here again, although previous studies reported no effect of client age on changes in alliance (28, 84), the findings of the current study suggest that client age may have a more complex effect on alliance. Specifically, older clients were found to benefit more from therapist FIS and were more negatively affected by their own interpersonal skills. Such nuanced understanding may facilitate progress toward personalization in both client treatment and therapist training.

Taken together, the explainable nature (a glass box vs. a black box) of the proposed framework of integrating explanation and prediction in psychotherapy may be instrumental in reaching a more nuanced understanding of the richness of clinical practice, where each predictor is not isolated from the others, but rather, interacts with them in complex ways in predicting the process and treatment outcome. Given the small sample size, the findings should be interpreted with caution, and serve mainly as a proof of concept demonstrating the potential utility of integrating explanation and prediction in computational psychotherapy science. Still, the results of these predictive models have the potential to assist in drawing a map of the factors contributing to the psychotherapy process and outcome and the complex interconnections between them. If replicated in future studies with large samples, the current findings offer instructive insights that expand the available literature on the predictors of psychotherapy process and outcome.

The most important limitation of the present study is the flip side of one of its main merits: its unique sample. Because of ethical considerations, maximizing variance in therapists results in minimizing variance in clients. To avoid restriction of range in therapist characteristics, we recruited a diverse set of treatment providers, who then provided counseling to a sample of individuals who had not actively sought treatment. For the same reasons, the effect of baseline characteristics was quite likely inflated. In the case of therapists who are more skilled in repairing alliance ruptures and who received appropriate alliance-focused training, it may be possible to rise above the deterministic view that the strengthening of alliance can be predicted based on the predispositions of the clients and bring about a real therapeutic change in the clients’ pre-treatment potential to form a strong alliance. In addition, since therapists represent both ends of highly skilled and unskilled communicators, this sample might have inflated effect sizes. Importantly, given the unique characteristics of the sample, it is questionable whether the results can be consistently generalized to psychotherapy settings. Another important limitation is that the small sample size restricted our ability to calculate therapist effects. Given the unique characteristics of the sample that were required, specifically, the richness of the variance and availability of baseline predictors (including therapist performance, as coded before the treatment, based on a standard evaluative task), we were limited by the available sample size. Future work on larger samples is critical. Thus, the current findings should be regarded as a proof of concept of our suggested framework of integrating explanation and prediction, rather than serving to inform clinical practice. In this work, we used a quasi-*R*^2^ estimate of Nakagawa and Shielzeth (67) because of the relatively simple data architecture and our interest in just the marginal explained variability. The approach suggested by Rights and Sterba (85) provides estimates with a better variance composition, taking into account various scenarios of the data and model structures. One should consider using these estimates.

Although the current proof of concept focused on client and therapist pretreatment characteristics, the proposed framework for integrating explanation and prediction in psychotherapy science can also be implemented on in-treatment data. Personalized treatment does not end with pretreatment clinical decision-making. Rather, ongoing tailoring is needed as well (86, 87). The literature on therapist responsiveness highlights the importance of ongoing tailoring of the treatment to the client. Effective therapists are responsive to client behaviors within the emerging context of the treatment (55). For example, observations of the many changing characteristics and behaviors of the client may prompt a therapist to use different interventions in the course of therapy (88). Likewise, the response of the client to a particular strategy may prompt the therapist’s next move, whether to stay the course or try something else—e.g., the therapist should not push an interpretation if the client responds to it defensively (55, 89). Therapeutic interactions exist in a constant loop of feedback and mutual influence (89). Using the proposed framework, ongoing data collection can be fed into the proposed models to support in-treatment decision-making processes (86). While the current proof of concept focused on pretreatment client and therapist characteristics, it is essential to note that the available data limited the construction of individualized models for each participant due to most variables having only one assessment per participant. Nevertheless, embracing idiographic approaches holds promise in potentially enhancing model predictability and optimizing treatment outcomes. Future studies adopting idiographic approaches are recommended to explore and leverage the benefits of constructing personalized models.

Explanatory models have contributed greatly to developments in the field of psychotherapy research, and today we know much more than we did 50 years ago about what drives therapeutic change and about the factors influencing it. This study demonstrates the great potential of the proposed approach to produce robust predictions of the process and outcome of treatment, offering a potential solution to the *p*-hacking and replicability problem. Such an approach is needed to answer questions in which future predictions are important, such as therapist selection, client prognosis to benefit from treatment, and so on. The present findings show how measures that have been used before in psychotherapy research can predict a large portion of the process (in this case, alliance) and outcome of psychotherapy, shedding new light on previously inconsistent findings.

## Data availability statement

The original contributions presented in the study are included in the article, further inquiries can be directed to the corresponding author.

## Ethics statement

The studies involving humans were approved by Institutional Review Board at Ohio University. The studies were conducted in accordance with the local legislation and institutional requirements. The participants provided their written informed consent to participate in this study.

## Author contributions

HF: Conceptualization, Writing – original draft, Writing – review & editing. SS: Conceptualization, Writing – original draft, Writing – review & editing. SZ-M: Conceptualization, Writing – original draft, Writing – review & editing. PG: Formal analysis, Conceptualization, Writing – review & editing. TA: Conceptualization, Resources, Supervision, Writing – review & editing.
