# Supplementary material for: Integrating exploration and prediction in computational psychotherapy science: proof of concept
Source: Front Psychiatry. 2024 Jan 12;14:1274764. doi: 10.3389/fpsyt.2023.1274764 (PMC10811256; doi:10.3389/fpsyt.2023.1274764)
Supplement: Supplementary file 1 [file Table_1.DOCX]

Table S1.

|  | 1 | 2 | 3 | 4 | 5 |
| --- | --- | --- | --- | --- | --- |
| 1. Psychological dysfunction | - |  |  |  |  |
| 2. Symptom severity | 0.89*** | - |  |  |  |
| 3. Interpersonal functioning | 0.70*** | 0.38 | - |  |  |
| 4. Client's social skills | 0.03 | 0.05 | 0.03 | - |  |
| 5. Interpersonal problems | 0.42 | 0.41 | 0.29 | -0.07 | - |

*Correlation Between the study variables*

*Note.* Psychological dysfunction was measured using the Outcome Questionnaire-45 (OQ-45). Symptoms severity and Interpersonal functioning are subscales of the OQ-45.

*** p<.001

Table S2.

|  |  | Mean (SD) | Range | IQR | RMSE (SD) | MAE (SD) |
| --- | --- | --- | --- | --- | --- | --- |
| Model 1  Predicting outcome | Target summary | 0 (1) | -3.21,2.197 | 0.94 | 0.73 (0.44) | 0.64 (0.38) |
|  | Prediction summary | 0.01 (0.78) | -1.81, 1.31 | 1.03 |  |  |
| Model 2  Predicting between-client alliance (C) | Target summary | 0 (1) | -2.12, 1.67 | 1.51 | 0.90 (0.59) | 0.82 (0.57) |
|  | Prediction summary | -0.08 (0.93) | -3.65, 2.57 | 0.94 |  |  |
| Model 3  Predicting between-client alliance (T) | Target summary | 0 (1) | -2.432, 2.199 | 1.18 | 0.62 (0.37) | 0.57 (0.34) |
|  | Prediction summary | -0.004 (0.83) | -1.07, 1.64 | 1.14 |  |  |
| Model 4  Predicting within-client alliance (C) | Target summary | 0 (1) | -1.96, 3.54 | 1.04 | 0.74 (0.41) | 0.66 (0.38) |
|  | Prediction summary | -0.01 (0.84) | -1.59, 3.96 | 0.49 |  |  |
| Model 5  Predicting within-client alliance (T) | Target summary | 0 (1) | -2.31, 2.05 | 1.06 | 0.70 (0.49) | 0.65 (0.49) |
|  | Prediction summary | -0.03 (0.80) | -1.43, 1.63 | 1.21 |  |  |

*Cross-Validation Model evaluation metrics*

*Note*. C- Clients' report; T- Therapists' report
